# Supplementary material for: A validation framework for neuroimaging software: The case of population receptive fields
Source: PLoS Comput Biol. 2020 Jun 25;16(6):e1007924. doi: 10.1371/journal.pcbi.1007924 (PMC7343185; doi:10.1371/journal.pcbi.1007924)
Supplement: S1 File — (DOCX) [file pcbi.1007924.s001.docx]

**Supporting Information**

# prf-Validation guide

The modular architecture of the testing framework is based on containers and the Brain Imaging Data Structure (BIDS) organization. Consequently means the tool runs on most platforms without compilation ([S1 Fig](#_30j0zll)). The synthesize-analyze-report pipeline is executed with a few command lines. The parameters that control the pipeline execution are specified by editing the three configuration files. More detailed instructions for installing the framework are maintained on a website ([https://github.com/vistalab/PRFmodel/wiki](https://github.com/vistalab/PRFmodel/wiki#how-to-install)).


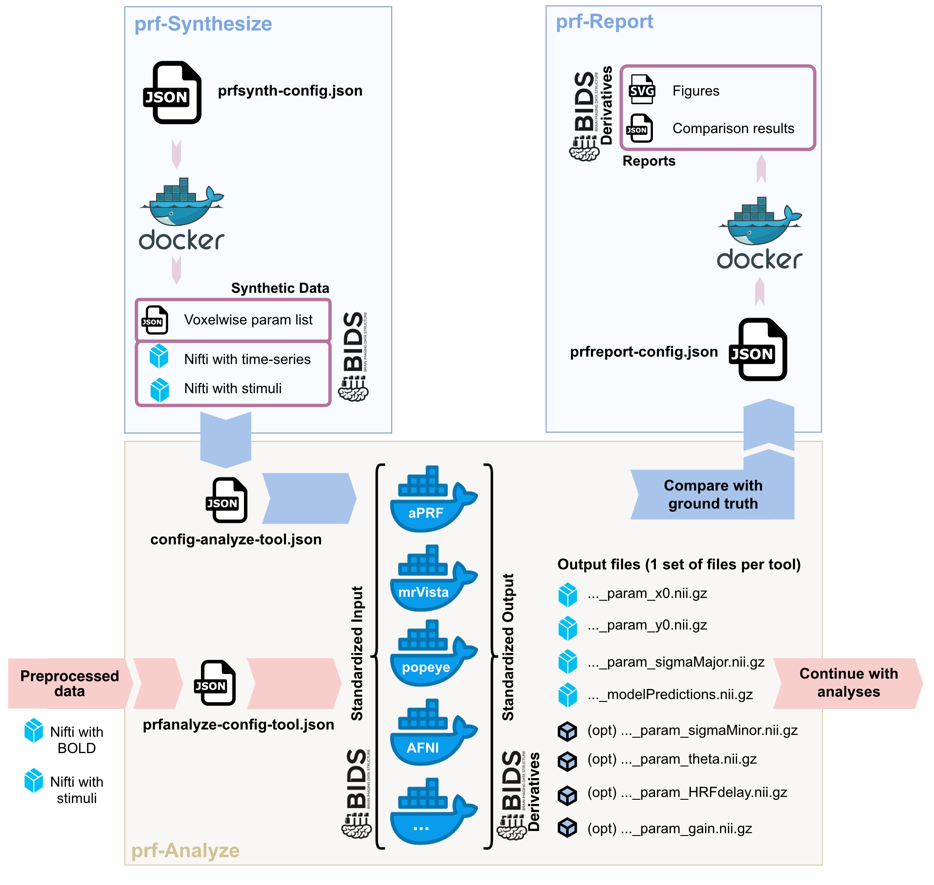


###### Figure A. Detailed working steps of the 3-element validation framework

*prf-Synthesize:* using a default json config file, allows the generation of a BIDS folder structure with the synthetic data and the parameters of every time series. *prf-Analyze:* Takes synthetic or real fMRI datasets in BIDS format, adapts them to different implementations of tools, and generates a BIDS derivatives output with uniformized output parameters. *prf-Report:* takes the synthetic dataset output from prf-Analyze and the voxelwise parameter definition with the ground truth from prf-Synthesize, and calculates goodness of fit reports.

## Installation

Docker must be installed in your system. The tools are installed with “docker pull” command lines. See installation instructions in the Github wiki (<https://github.com/vistalab/PRFmodel/wiki#how-to-install>).

## prf-Synthesize

The prf-Synthesize container is invoked by the command ‘run_prfsynth’ or with a direct ‘docker run’ call. That command takes two arguments: (a) the name of the configuration file , and (b) the full path to the output directory. The output file names are assigned by the container. A default configuration file can be created by running the container with the arguments ‘empty’ and the path to the output folder (copy and paste examples can be found in <https://github.com/vistalab/PRFmodel/wiki>). Using a text editor, the user modifies the json file, specifying the parameters for the synthetic BOLD time series. Detailed instructions on how to edit the file can be found in the GitHub wiki (<https://github.com/vistalab/PRFmodel/wiki/prf-Synthesize:-how-to-edit-json-file>). After the json file has been edited, the user runs the Docker container again to create the synthetic data in the output folder.

The container is designed to create a grid of all combinations of the configuration parameters. For example, if the configuration file includes two center locations for x and y, say 0 and 5, the container generates 4 synthetic BOLD series with centers at [0,0], [0,5], [5,0] and [5,0]. If two HRFs are specified in the configuration file, say ‘popeye_twogammas’ and ‘afni_spm’, the number of synthetic BOLD time series doubles to 8; the four center locations times two HRFs. If the configuration parameter ‘repeats’ is set to 100, 100 noisy samples are generated for every parameter combination. The documentation in the GitHub wiki contains a more detailed description.

Upon successful completion the container creates a new folder called ‘BIDS’ in the output directory. The files are organized following the BIDS guidelines. The files written out by prf-Synthesize are used by the analyze and report elements of the framework.

## prf-Analyze

The prf-Analyze container is analogous to prf-Synth. It is invoked by the command ‘run_prfanalyze’ or a direct ‘docker run’ call. Check the wiki in <https://github.com/vistalab/PRFmodel/wiki> for copy+paste examples and detailed information.

## prf-Report

The usage is analogous to the other two containers. We run it with the ‘empty’ parameter to obtain the default configuration json file. Then we run it with the edited config file to perform the analysis. See detailed instructions (<https://github.com/vistalab/PRFmodel/wiki/prf-Report:-how-to-edit-json-file>) about editing the config file. The output results are saved in the BIDS derivatives folder as well.

# Visualization and customization of the main elements

The Docker containers were implemented to simplify running the code on most platforms. If one desires to examine the components of the calculations one-by-one and check individual results or hypotheses, it is also possible to run the code manually within the Matlab command line interface. Download and set-up the code repository (<http://github.com/vistalab/prfmodel>) and follow the online instructions. Check the guide in the wiki for further detail

(<https://github.com/vistalab/PRFmodel/wiki/Visualization-and-customization-of-the-main-elements-in-Matlab>).

# Public pRF implementations

We maintain a list of public pRF analysis tool implementations here (<https://github.com/vistalab/PRFmodel/wiki/Public-pRF-implementations>).

# pRF analyses with additional location and size parameters

We provide several low noise simulations to verify that the main effects hold true independent of the specific center and size (Figures S2-S5). the hrf dependence affects the recovery of the size, meanwhile the median pRF center location is accurate.

#
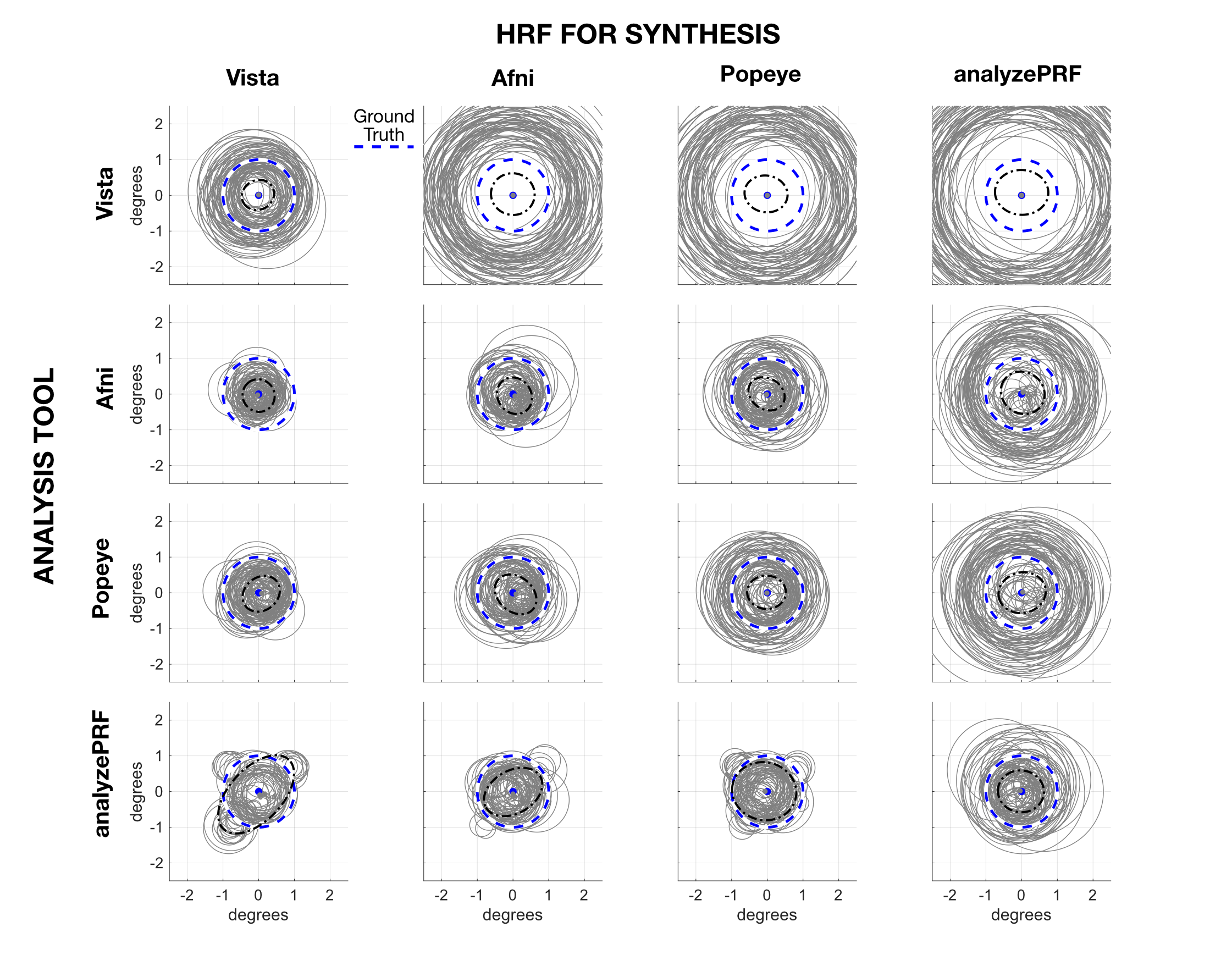


###### Figure B. Noise analysis and HRF dependence (x=0, y=0, radius=1deg, fov=20)

Each column shows the HRF used in pRF-Synthesize to simulate the BOLD time series. The prf-Synthesize tool created BOLD signals with the typical noise level (low level, see Figure 4) and a circular RF centered at (0,0) deg and 1 deg radius (dashed blue circle). Each row corresponds to the pRF-Analyze tool, with its default HRF, that was used to analyze the data. The gray circles show the central 90% size percentiles of the estimated RFs. The central ellipsoid includes 90% of the estimated centers (dashed-dot, black). The central black dot shows the median center location. The HRF used in the synthesis matches the HRF assumed in the analysis in the plots along the diagonal (dashed black rectangles). Above the diagonal, the synthesis HRF is narrower than the analysis HRF; below the diagonal the opposite is true.


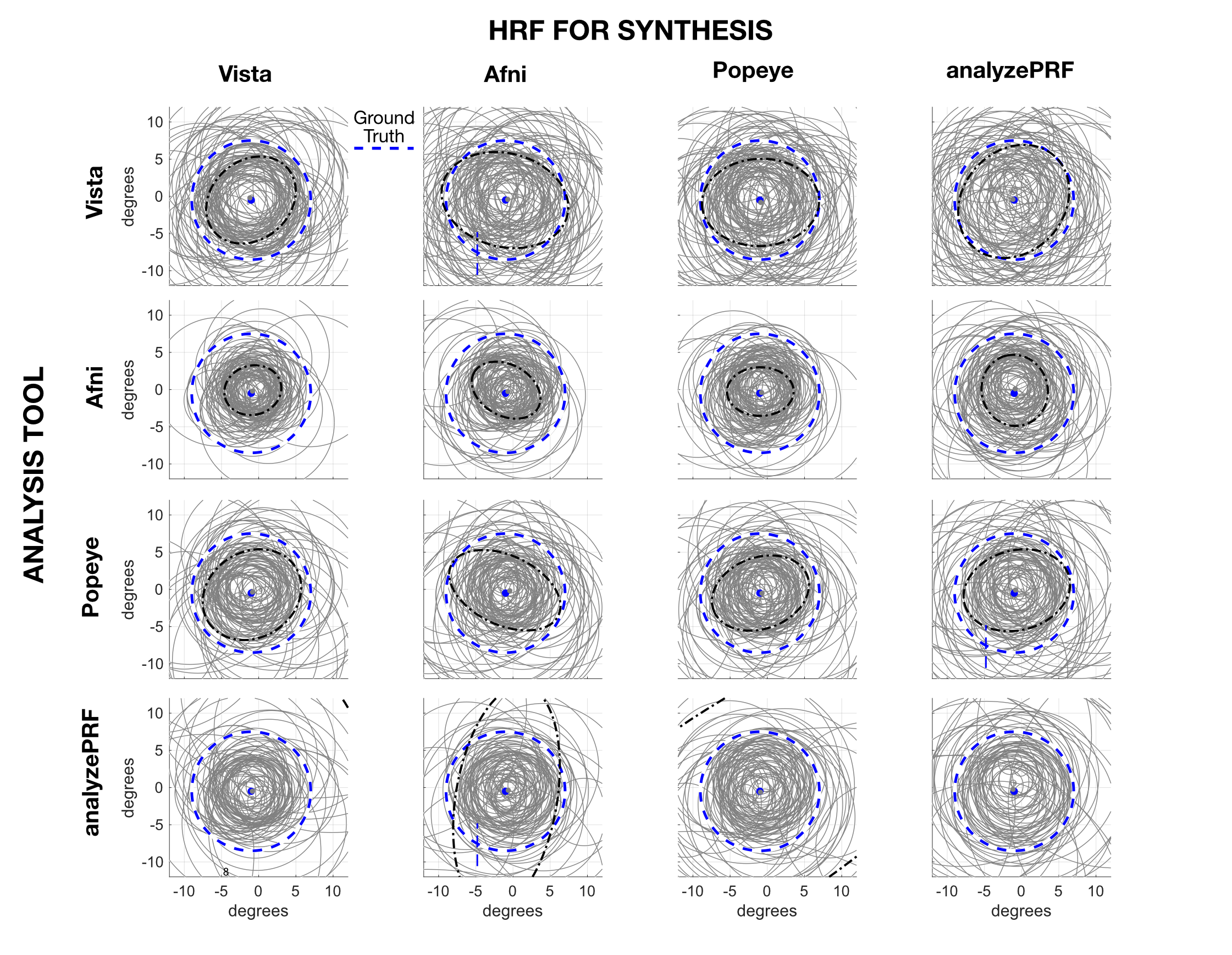


###### Figure C. Noise analysis and HRF dependence (x=-1, y=-0.5, radius=8deg, fov=20)

Other details as in S2 Fig.

#

#
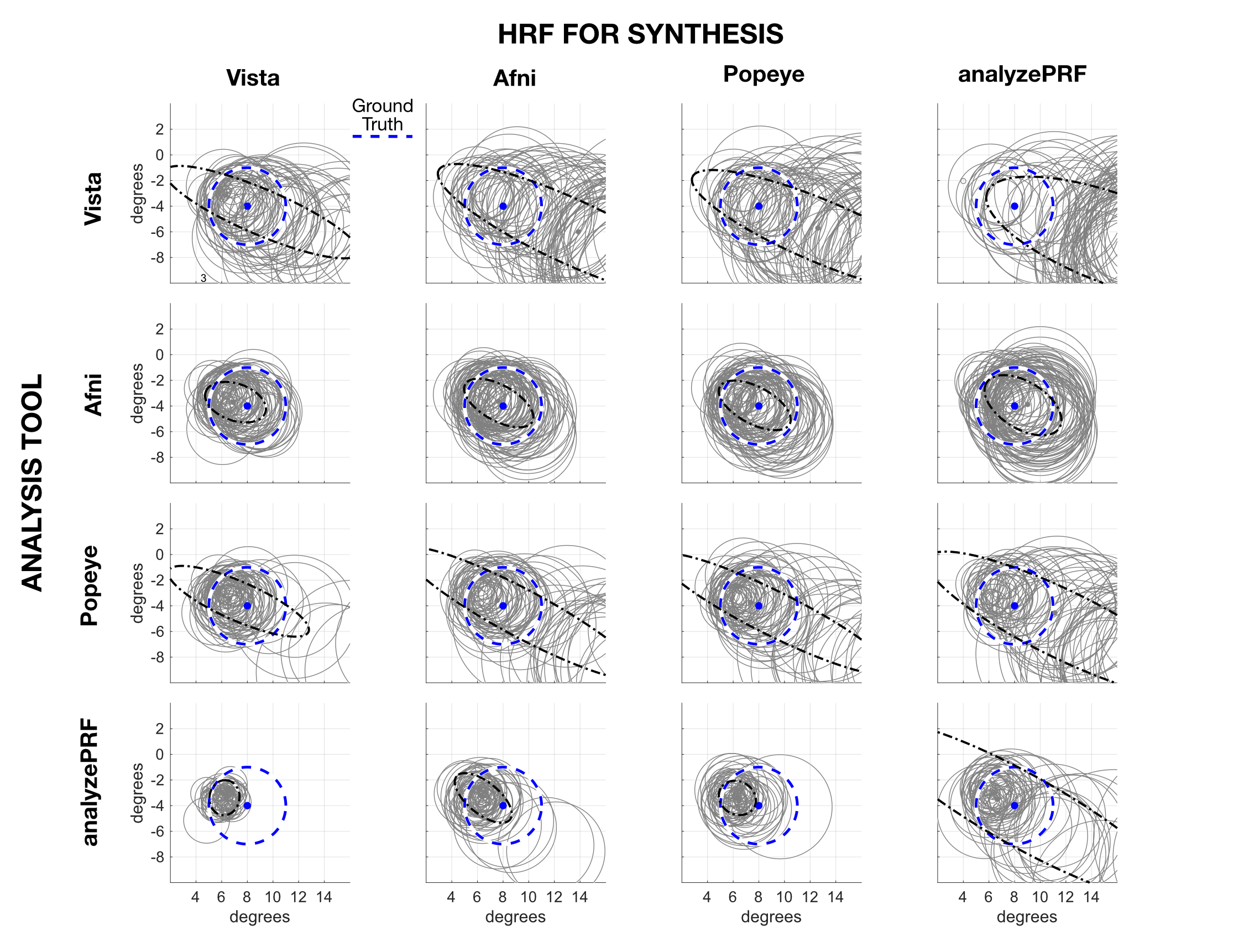


###### Figure D. Noise analysis and HRF dependence (x=8, y=-4, radius=3deg, fov=20).

###### The position and size in this case places some of pRF outside of the field of view, which is a particularly challenging case. Other details as in S2 Fig.

#

#
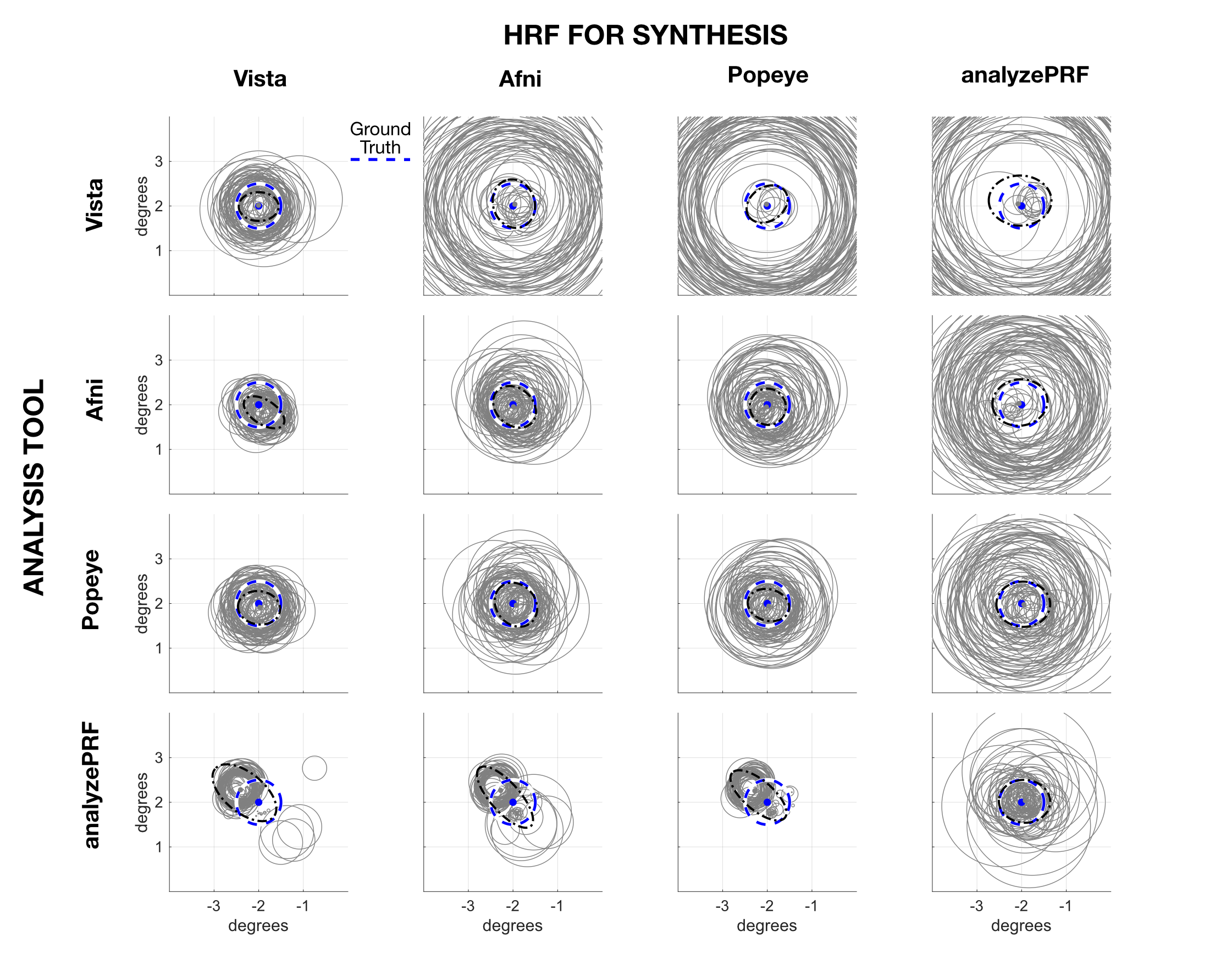


###### Figure E. Noise analysis and HRF dependence (x=-2, y=2, radius=.5deg, fov=20)

###### Other details as in S2 Fig.
