# Supplementary material for: A validation framework for neuroimaging software: The case of population receptive fields
Source: PLoS Comput Biol. 2020 Jun 25;16(6):e1007924. doi: 10.1371/journal.pcbi.1007924 (PMC7343185; doi:10.1371/journal.pcbi.1007924)
Supplement: Attachment — Submitted filename: Response Letter.docx [file pcbi.1007924.s002.docx]

# RESPONSE LETTER

## Reviewer #1:

This study contains a worthwhile effort to validate the estimates of population receptive field (pRF) parameters with a range of common software packages used in the literature. I welcome the authors' intention because such validation is important and desperately needed. One would certainly hope that the authors of previous pRF analysis tools already performed such simulations on their methods in-house, and in fact several authors have in fact published such validations, which would deserve some more discussion. Nevertheless, a more comprehensive effort is certainly very timely, considering the popularity of these methods now. I would be very interested to extend the authors' approach to our analysis tools (although I am not sure I have the technical knowledge to use the Docker approach presented here without further assistance). In general, I very much like the authors' suggestion of using such an approach to guide the most effective experimental design of future studies.

We thank the reviewer for these comments, and we agree with them.

Description of changes:

We expanded our discussion of papers in which authors reported software validation tests of their software. These are discussed on Page 5, Lines 109 to 112 (P:5, L:109-112). We also explicitly discuss different types of validation (P: 5, L:114-139).

On a personal note, we will be glad to assist with the Docker implementation.

That said, I think there are a number of limitations to the study presented here and this could make this study more comprehensive and increase its impact on the field:

1. As far as I understand, the simulated BOLD time series are based on Kay's analyzePRF methods. As such it doesn't seem very surprising that this method seems to reproduce the ground truth most accurately in the noise-free data (Figure 5). This design seems asymmetric. If generating the pRF time course using the AFNI method, will this result in more precise estimates for AFNI?

**Response:**

No, we regret the confusion. Only the stimulus was generated with the Kay source code. The time series for the analyses were created using the synthesis code we implemented for this project. The time series computation involved only using the symmetric Gaussian pRF, convolution with one of the HRFs and in some cases additive noise. The specific HRF is documented in each of the figures. The noise model is described in Figures 3 and 4.

**Description of changes:**

We edited the section describing how we used Kay’s code in the Methods | Stimulus section (P:9, L:248-254). We clarified that we only used that code to create the binary space-time stimulus.

2. The differences between the methods also deserve more discussion. To my knowledge, the AFNI method is purely based on an extensive grid search whereas in mrVista this varies between studies (although the standard method includes an optimisation step). I assume default parameters were used for each method but it would be useful to compare this, perhaps in the form of a table.

**Response:**

There are substantial differences between the model implementations and also between the parameter sets that control these models. In fact, many of the parameters are incommensurate. For example, the analyzePRF implementation defaults to using the compressive spatial summation model which is not implemented in AFNI. Some models include the option of searching for an HRF, while others do not include this option. There are other differences between the models of this magnitude.

To enable a comparison between the models we examined a condition that all of the models address: a linear model with a circularly symmetric Gaussian receptive field. Even to make this comparison it was necessary to modify the analyzePRF code to fix the compressive parameter to one.

Given the complexity of the implementations - and several of the implementations have more than a dozen parameters - we think it is best for the reader to read the authors’ own descriptions, either in publications or in the software. It is beyond our ability to explain and justify the many choices made in each of the implementations, and in a sense that is the point of this paper.

**Description of changes:**

We now discuss these issues at greater length in the text following Figure 5 (P:14, L:383-390).

3. It is unclear to me why only one pRF at one location and size was chosen for the assessments. Surely the situation might differ considerably across different ground truth eccentricities or with different ground truth pRF sizes (which in turn would probably have implications for different visual areas)?

**Response:**

The findings reported in this paper hold true across different ground-truth pRF eccentricities and sizes that we tested. There are biases related to the HRF choice and the size of the bias depends on specific eccentricity/angle. In all cases the pRF size is influenced more than the center position. The relative accuracy of the center and the large uncertainty in the size is evident in the simulations reported in the paper.

**Description of changes:**

In response to the Reviewers comment, we added four new Supplementary figures using different combinations of location and size parameters. We refer to these Supplementary figures in the main text (Page 15, Line 436-437), before Figure 7 (and in the caption of Figure 7).

4. Why did you choose an oriented 2D Gaussian chosen as the underlying pRF model for the simulations? As far as I can tell, the most commonly used pRF model is a circularly symmetric 2D Gaussian. Unless I misunderstood, it also seems like the analysis of the data you present reflects a circularly symmetric model and the data analysis appears to be based on a single example pRF that is also symmetric (see point 3).

**Response:**

We presented the general model, which includes the elliptical and the difference of Gaussians, in the equations on page 8 and 9. In the analyses we report, however, we always tested with the two equal sigmas (circularly symmetric) and no Gaussian surround (DoG).

**Description of changes:**

We added a clarifying note about the generality of the implementation but the specialization in this paper. This note is placed after we describe the equations defining the general model (P:9, L:244-246). There is also a change in the Discussion (P:21, L:585-589) in which we address this issue.

5. I may have missed this but is the data using the difference-of-Gaussians (DoG) pRF model actually used in any of the analyses? If not, then it might be sensible to remove this (you could say that this is possible to synthesise if desired). I am also not clear as to why this DoG model seems to have the same sigma (pRF size) parameters for the positive and negative components. For the DoG models implemented by Zuiderbaan et al (2012, J Vis), Schwarzkopf et al (2014, J Neurosci), or Anderson et al (2017, J Neurosci) the negative component is typically a larger surround and thus would have a larger sigma than in positive, central component (and these models did not use oriented, non-circular pRFs).

**Response:**

This is connected to the Reviewer’s Comment (4). We include the description of the general models because the tool is capable of doing these analyses. But we now make clear that the analyses included in the paper did not make use of the ability. We plan to make use of these features in the future.

**Description of changes:**

See changes for Comment #4.

6. The analysis about sweep duration and stimulus randomisation doesn't appear until the Discussion. I feel this belongs into the results section. It makes sense to me that the randomised designs have a weaker signal-to-noise ratio and this matches what Binda et al (2013, J Vis) observed but then again we found in Infanti & Schwarzkopf (2020, NeuroImage) that randomisation did not impact signal-to-noise all that much - although this could represent a ceiling effect because we only estimated polar angles in those experiments.

**Response:**

We agree that it fits better into the results.

**Description of changes:**

We moved the ‘HRF dependence’ section from ‘Discussion’ to ‘Results’.

7. You suggest that polar angle and eccentricity errors between the methods are negligible. This certainly matches the findings based on the reliability from Senden et al (2014, PLoS One), van Dijk et al (2016, NeuroImage), and Benson et al (2018, J Vis). I think these findings should be discussed in the context of these reliability studies. Of course, the aforementioned caveat applies that all the present results only speak to one simulated pRF of with x,y position 3,3 and radius 2 deg. It would be important to quantify this across a plausible range of pRF parameters.

**Response:**

We agree that these papers should be discussed.

**Description of changes:**

- We added the references into the manuscript (see P:5, L:118 and P:20, L:573).
- We produced other combinations of analyses and generated different versions of Figure 7 in the Supplemental Materials (See changes for Comment #3).

Minor points:

Line 140: "is comprised of" - I'm probably pedantic and also realise that this is a grey area - but I'd change this to "comprises" or "consists of"

**Description of changes:**

Fixed. Thanks.

Line 145: typo - "noise-free" should be "synthetic"

**Description of changes:**

Fixed. Thanks.

Line 261: "summer 2019" - while I know what you mean this is ambiguous unless you're a Flat Earther and don't believe in the existence of the southern hemisphere. Change to "mid-2019"?

**Description of changes:**

Ah, so there really is a Southern Hemisphere. Good to know. We had heard rumors of smart people down there. Fixed.

Line 284: In all the figure captions you state that the radius SD of the simulated pRF was 2 deg. This matches also the ground truth shown in the figures themselves. But here in the text there appears to be a typo as it is reported as 1 deg.

**Description of changes:**

Fixed. Thanks.

Jargon and figure labels: these could be made more consistent. For example you seem to move between signal percent change, percent signal change, and relative amplitude - sometimes even within the same figure.

**Description of changes:**

Thanks, we now use percent signal change throughout.

Citations for pRF models: You should probably cite Zuiderbaan et al (2012, J Vis) for the DoG pRF models (although this is a simpler and somewhat different model than you use here - see point 5). For the oriented pRF model you should probably cite Silson et al (2018, J Neurosci). As mentioned earlier, I think the most widely used model is the circularly symmetric Gaussian model first presented by Dumoulin & Wandell (2008).

**Response:**

We now cite and discuss the Zuiderbaan et al paper. The Silson et al claim of anisotropic pRFs is worth exploring in more detail, and we feel that introducing the topic here would distract from our main point. As the other Reviewer points out, this paper is mainly about the validation framework. Further work will bring us deeper into the domain specific ideas (DoG, anisotropic, nonlinear models).

**Description of changes:**

See response to Comment #4.

Reference list citation 28: My name is misspelled in that reference as "Sam Schwarzkopf D" when the correct reference should read "Schwarzkopf DS". I've noticed this before and may have something to do with how reference managers parse the author list. Sorry…

**Description of changes:**

Fixed. Thanks.

Reviewer #2

This paper presents a general computational framework for evaluating neuroimaging software, with both computational reproducibility and validity.

The authors demonstrate this framework by applying it to population receptive field (pRF) methods for fMRI, and they present initial findings comparing different pRF tools using this framework.

Two highlighted features of the framework are the containerised architecture (with BIDS implementation) which ensures reproducibility, and the generation of synthetic data with known ground truth to evaluate validity.

Within the pRF context, the authors report that this validation framework allowed them to identify and report issues with some pRF tools, and a significant dependence on the HRF model used in different implementations.

Furthermore, it is proposed that the framework can be extended to evaluate other neuroimaging algorithms.

I acknowledge the importance and need for such frameworks and their utility for comparing across different neuroimaging tools within a common validation framework.

The paper is clear, and mostly well written. However, I am confused about the emphasis of this paper. Initially, from the title, abstract and introduction, I was under the impression that this paper was describing a general tool that could be applied to many neuroimaging contexts, but which would be demonstrated in the pRF context. However, through the methods results and discussion, the focus seem to shift heavily towards the pRF specific application.

I am left unclear if this is a general framework that is demonstrated in a pRF application, or a pRF framework that could, but has not been, adapted to be more general…. I appreciate that this may be a subtle difference, but to many readers it will be an important distinction.

If it is the former, then much more detail is required about how the framework would need to be adapted for other neuroimaging contexts, and how much effort that would be.

If the latter, then I suggest you embrace this as a pRF validation tool and downplay the more general sounding descriptions in the earlier parts of the paper, particularly the abstract and title. You should also promote the pRF results, which figure prominently in the discussion, to the abstract. In this form, you will need to take the evaluation of the pRF tools to its full conclusion; as you currently only describe initial findings.

**Response:**

We appreciate the Reviewer’s point. It is our view that we describe both a general framework and then demonstrate the realizability of the framework through a complete implementation for one case. We feel secure that we included everything needed for such a framework because the pRF implementation forced us to think through all the requirements. The application part is important to illustrate how a complete framework would work, and what kind of results could come from applying such a framework.

Even so, we understand the Reviewer’s suggestion. We agree that it would be helpful to the reader if we more clearly distinguish between the Validation Framework and the application to pRF. Changes we made based on the Reviewer’s comments should help the reader understand the distinction we intend between the main point (Validation Framework) and the demonstration of the framework in the pRF application.

**Description of changes:**

We substantially changed and reorganized the Abstract, Introduction, and Discussion.

- Abstract (P:2, L:37-48)
- Introduction: reorganization and additional paragraphs (P:4-6).
- Discussion: reorganization and new additional paragraphs (P19; P:20,L:564-570).
- New Conclusion section in P:24.

Major issues:

Other than the emphasis, which I have already discussed, I have no other major issues with this paper.

Minor issues:

I think that the limitations section could benefit from some discussion of ground-truth versus truth, and generalisation. As this framework is dependent on synthetic data, the risk is that software will be optimised towards the ground-truth of synthetic data, which could reduce its generalisation to “real” data. Perhaps it would have been worthwhile performing evaluation with real data alongside the synthesised data, especially considering that the x-Analyze model already supports this.

**Response:**

We appreciate the reviewer’s concern that tools are made to be used with real data. If these pRF tools were entirely new, we would apply them to real data here. However, the tools we incorporated into our validation pipeline have already been applied to real data data in dozens of published papers. Some of those papers test tool validation in a different sense from what we study here, specifically whether results are repeatable or lead to a high variance explained. The advance here is the ability to test ground truth, which is not known in real data. The ability to generate appropriate synthetic data is critical to our enterprise. One of the things we learned in the process is that there is great value in synthetic data - even though that is not realistic data. Similarly, there is value in generating synthetic data that is more noisy than one would normally accept.

**Description of changes:**

In response to the Reviewer’s point, we expanded the Discussion to explain the role of synthesis and its connection to developing computable models of the experiment. We also added a new section in the Introduction called ‘Types of validation’ (P:5,L:115).
